# Defining the “Correlate(s) of Protection” to tick-borne encephalitis vaccination and infection – key points and outstanding questions

**DOI:** 10.3389/fimmu.2024.1352720

**Published:** 2024-01-22

**Authors:** Rahel Ackermann-Gäumann, Phung Lang, Kyra D. Zens

**Affiliations:** ^1^ Microbiologie, ADMED Analyses et Diagnostics Médicaux, La Chaux-de-Fonds, Switzerland; ^2^ Swiss National Reference Center for Tick-transmitted Diseases, La Chaux-de-Fonds, Switzerland; ^3^ Epidemiology, Biostatistics and Prevention Institute, University of Zurich, Zurich, Switzerland; ^4^ Institute for Experimental Immunology, University of Zurich, Zurich, Switzerland

**Keywords:** tick-borne encephalitis (TBE), correlates of protection, cellular immunity, humoral immunity, vaccination, orthoflavivirus

## Abstract

Tick-borne Encephalitis (TBE) is a severe disease of the Central Nervous System (CNS) caused by the tick-borne encephalitis virus (TBEV). The generation of protective immunity after TBEV infection or TBE vaccination relies on the integrated responses of many distinct cell types at distinct physical locations. While long-lasting memory immune responses, in particular, form the basis for the correlates of protection against many diseases, these correlates of protection have not yet been clearly defined for TBE. This review addresses the immune control of TBEV infection and responses to TBE vaccination. Potential correlates of protection and the durability of protection against disease are discussed, along with outstanding questions in the field and possible areas for future research.

## 1 Introduction

Defining the so-called “correlates of protection” against a disease, namely which immune subsets are capable of consistently protecting individuals from illness and at which levels, is critical not only for monitoring responses to vaccination, but also for assessing susceptibility to disease in the population and developing immunization strategies. Tick-borne Encephalitis (TBE) is a severe, vaccine-preventable disease of the Central Nervous System (CNS) caused by the tick-borne encephalitis virus (TBEV) and transmitted to humans primarily through the bite of infected Ixodid ticks. TBE is typically caused by infection involving one of three TBEV subtypes, namely the European, Siberian, and Far Eastern subtypes, transmitted primarily by *Ixodes ricinus* (European subtype) and *I. persulcatus* (Siberian and Far Eastern subtypes) ticks, with the distribution of viral subtypes reflective of the geography of their respective tick vectors [reviewed by (1–3)]. In addition, two other viral subtypes, Baikalian (4) and Himalayan (5), have been recently described. TBEV is widespread throughout Central, Eastern, and Northern Europe as well as parts of Asia with between 10,000-15,000 cases reported annually [reviewed in (1, 3, 6)]. These estimates, however, likely represent just a subset of the total disease burden as the sometimes mild, or unspecific nature of the disease most certainly contributes to undertesting and underreporting of cases. In addition to preventing tick bites, active immunization is the most important protective measure against TBEV infections. Europe uses two of the six licensed vaccines. The standard immunization schedule for both of these vaccines includes three doses, followed by regular boosters to maintain protection [reviewed in (7–10)].

The immune responses which protect individuals against disease represent a complex interplay between many distinct cell types at various times and over different locations. Innate immunity comprises the “first line” defenses following pathogen exposure, acting rapidly and broadly to protect against invaders. Adaptive immune responses, comprised by both humoral (i.e. antibody), and cell-mediated (i.e. T cell) responses, take more time to be established as they require the initial activation of the innate immune system, but provide highly-specific protection against invading pathogens, and further offer immune memory – a subset of cells which are maintained long-term (up to decades), and provide rapid protection upon later re-exposure to the same pathogen. These memory immune responses form the basis for vaccination as well as the correlates of protection. Here we review our current understanding of the immune responses to TBEV infection and TBE vaccination, focusing on potential correlates of protection.

## 2 TBEV transmission and early and innate immune responses to infection

Small mammals serve as the natural reservoir for TBEV with humans acting only as “dead end” hosts. While TBEV is transmitted primarily through tick bites, approximately 1% of cases occur via consumption of unpasteurized dairy products produced from the milk of viremic animals [alimentary transmission, reviewed in (3, 6)] and rare cases of transmission via organ or blood donation have been documented (11, 12). Within the tick vector, the virus is thought to reside within the salivary glands and is thought to be transmitted, via saliva, in the first several minutes following a bite (13). Transmission of TBEV is further facilitated by factors within the tick’s saliva [(14) reviewed in (15)] which contains components that suppress both local innate responses, as well as the initiation of adaptive immunity [reviewed in (16)].

Following infection, an estimated 70% of TBEV exposures are asymptomatic [reviewed in (17–19)]. This is, however, likely a substantial underestimation. Recent nationwide seroprevalence estimates from Switzerland, for example, indicate that approximately 5% of the unvaccinated population is seropositive for TBEV (20), although the average annual incidence is only 3-5 cases/100,000 individuals; approximately 1000-fold lower (21), suggesting that further studies are needed to better understand the true burden of infection.

Of individuals which do go on to develop symptomatic illness, approximately 70-80% experience a single phase of influenza-like illness after an incubation period ranging from 2-28 (generally 7–14) days following tick bite. The incubation after foodborne infection is generally shorter, around 4 days. Initial illness typically lasts approximately 1 week (1-10 days) and is characterized by non-specific symptoms such as fatigue, fever, headache, and myalgia. The first phase of disease is followed by clinical amelioration or an interval without any symptoms for up to 1 week (range 1-31 days). Around 20-30% (up to 46%) of patients experiencing the first clinical phase go on to develop a second phase of TBE characterized by CNS involvement [reviewed in (3, 6, 7, 22)]. In adults, symptoms of CNS disease include meningitis, encephalitis, myelitis, radiculitis, or any combination of these. TBE caused by the European viral subtype presents as meningitis alone in roughly 40% of cases and includes encephalitis in 55% of cases. The most severe forms of disease include myelitis and occur approximately 5% of the time (23–26). These manifestations are often milder in children, though severe disease does occur [reviewed in (27)]. Myelitis can lead to paresis of the extremities, or of the respiratory muscles requiring artificial ventilation. Following CNS disease, permanent sequelae occur in 30-50% of affected individuals. Sequelae range from mild (approximately 30%), to moderate (approximately 60%), to severe (approximately 10%) with the severity of sequelae correlating with the severity of acute disease. Death occurs in 0.5-2% of clinical cases and tends to depend on age (23–26). Fatality rates tend to be higher following disease caused by the Siberian or Far Eastern viral subtypes and special disease forms, such as chronic progressive disease and a hemorrhagic form, have also been associated with these subtypes [reviewed in (7)].

### 2.1 Infection in the skin and early immune control

**Figure 1:**
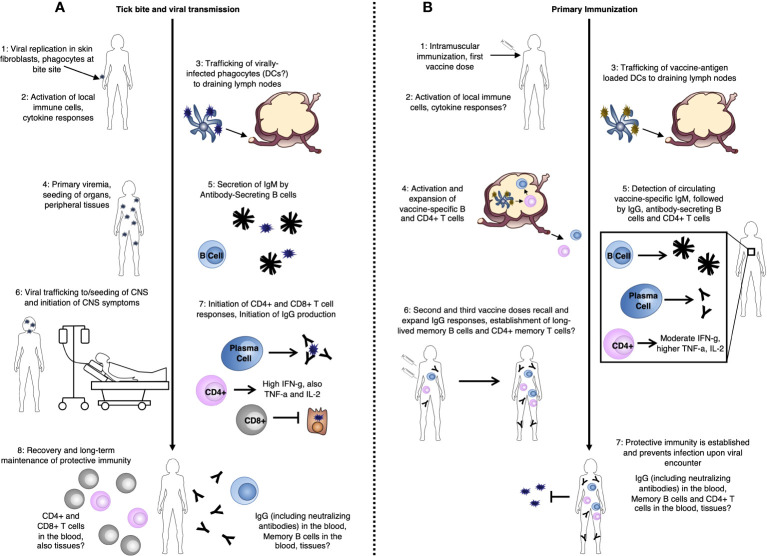
TBE-specific immune responses **(A)** after tick bite-mediated transmission of the TBE virus, and **(B)** after TBE vaccination.

The innate immune system comprises the earliest defenses against viral infection and is particularly important in “naïve” hosts that have not yet been exposed to a particular pathogen and developed protective adaptive immune memory. TBEV belongs to the genus *Orthoflavivirus*, which also includes the clinically-relevant, arthropod-borne viruses Dengue, West Nile, Yellow Fever, and Zika (3, 6, 28) and the early immune responses to TBEV infection share many features with these viruses (29). Innate immunity can be divided into an intrinsic intracellular response elicited by viral infection, and an innate extracellular response mediated by specialized immune cells [reviewed for TBE in (7)]. Innate immune recognition of pathogens relies on the host’s expression of pattern recognition receptors (PRRs), which detect conserved moieties expressed by potential pathogens. Following exposure to TBEV-infected ticks, gene expression analyses have demonstrated that local skin inflammatory responses already begin within the 1-3 hours of attachment of [(30–32), **Figure 1A**].

#### 2.1.1 Viral recognition

PRRs important in the detection of RNA viruses, in general, include Toll-Like Receptors (TLRs) and Retinoic Acid-Inducible Gene I (RIG-I)-Like Receptors (RLRs), including RIG-I and Melanoma Differentiation-Associated protein 5 (MDA5). PRR activation leads to signaling cascades which result in the activation of the Interferon (IFN) regulatory factor 3 (IRF-3) signaling pathway and subsequent production of IFN. While the function of TLR signaling in protection from TBEV infection is less clear, with potential involvement of TLR-3 [reviewed in (33)], roles for RIG-I and MDA5 in the innate immune recognition of TBEV proteins, including non-structural protein 5 (NS5) have been demonstrated (31). This results in an early immune response dominated by type I IFN (IFN-α and IFN-β), which seems to be the key mediator of protection during initial stages of infection in both *in vitro* and *in vivo* models (34, 35), similar to many other viral infections. Consistent with this, mice lacking the IFN-α/β Receptor (IFNAR), a key type I IFN receptor, are unable to control TBEV infection and studies of polymorphisms in innate immune response genes in patients have identified polymorphisms in the interferon-induced antiviral proteins oligoadenylate synthetase 2 (OAS2) and 3 (OAS3) which may predispose individuals for the development of clinical TBE (36). Of key importance, however, is that local, early immune responses which control the virus at this stage before further spread could prevent the establishment of later clinical disease (**Figure 1A**).

#### 2.1.2 Local dendritic cell responses

DCs represent a group of cells with a range of functions including acting as a major source of type I IFN during viral infection and playing critical roles in antigen presentation and activation of adaptive immune responses. After TBEV is transmitted, skin-localized DCs are among the first cell types to be infected and they likely play roles in viral trafficking (**Figure 1A**). Thus, DCs represent a key cell type in the TBEV life-cycle within the human host. Infection of DCs *in vitro* with Langat virus (LGTV), an attenuated member of the TBE serogroup, inhibits type I IFN signaling and reduces IL-12 production – a key activator of type 1 adaptive immune responses (37). Furthermore, *in vitro* infection of DCs with distinct TBEV strains results in distinct functional capacities, impacting later activation of CD4+ T cells (38). This is further supported by recent work demonstrating differential activation of IRF-3 and Protein kinase B (PKB/Akt) by high and low virulence TBEV strains (39). Together, these finding suggest potential TBEV strain-specific differences in the activation of subsequent adaptive immune responses. Additionally, higher infectious doses in mice led to delays in DC activation and IFN production, and impacted viral spread to the CNS, indicating the importance of the initial infectious dose on downstream immune responses also dependent on DCs (38). This is perhaps not surprising given the key role of DCs as the interface between innate and adaptive immune responses.

### 2.2 Primary viremia and seeding of peripheral tissues

In the absence of early local immune control within the skin, the virus next traffics to the draining lymph nodes (**Figure 1A**). This process is not yet well understood, but it is thought that the migration of virally-infected phagocytes or DCs from the skin following activation, as described above, may play an important role (40). This initial trafficking occurs during the asymptomatic incubation phase of illness [2-28 days post-viral exposure (3, 6, 22)]. Once within the lymph nodes, the virus undergoes further rounds of replication, eventually seeding peripheral tissues. During this viral expansion into the periphery, the host experiences a period of primary viremia [3-38 days post viral exposure (3, 6, 22, 41, 42); **Figure 1A**]. Consistent with other studies assessing the early immune response to TBEV infection, work in a mouse LGTV model has demonstrated that the type-I IFN response is critical for control of this initial viral replication and systemic viral spread (43). Otherwise, relatively little is known about potential mechanisms for viral control at this primary viremic stage of infection. However, due to the delayed kinetics of the initiation of adaptive immune responses, antibody and T cell responses likely do not yet play an important role in the protection of “naïve” hosts at this stage.

### 2.3 Secondary viremia and CNS disease

In a biphasic disease course, CNS symptoms occur anywhere from 4 to 68 days post viral exposure (3, 6, 22). TBEV is neurotropic – preferentially infecting cells of the nervous system. TBEV replication, for example, is 10,000-fold higher in human neuronal cells compared to epithelial cells (44). The ability of the virus to cross the blood brain barrier and invade the CNS is the root cause of human disease. The route by which CNS seeding occurs, however, is not well-understood, though breakdown of the blood brain barrier (BBB) does not appear to be necessary for TBEV entry into the brain (45, 46). The virus is generally no longer present in the blood once CNS involvement becomes clinically apparent.

Patients typically present to the clinic only after the occurrence of clinical CNS illness, and much of what is known about immune responses to TBEV in humans has been observed in the context of CNS disease. Several studies have reported a host of cytokines to be upregulated in the blood of TBE patients including Chemokine (C-C-motif) Ligand (CCL)5, CCL7, Chemokine (C-X-C-motif) Ligand (CXCL)10, CXCL11, CXCL13, Tumor Necrosis Factor (TNF)-α, Interferon (IFN)-γ, Interleukin (IL)-1 α, IL-6, IL-15, and IL-18, among others (47–53), though a “TBE-specific” cytokine profile, which could be useful for diagnostic purposes, has not been defined. In general, the response appears to be heterogenous but consistent with a type 1 immune response, typical of viral infections. Importantly, cytokine-mediated trafficking promotes the entry of immune cells into the brain, which may contribute to immunopathology observed during severe infection in animal studies (46). In TBE patients, increased levels of CCL5 (47) and CXCL10 (47, 50) in the cerebral spinal fluid (CSF) may recruit T cells [via expression of CCR5 (47) CXCR3 (50)] into the brain during disease. Similarly, TBEV-infected mice demonstrate increased levels of CXCL10 in the serum and brain during infection (54). Strong cytokine expression in the brain, coupled with very low neutralizing antibody responses, has been linked to enhanced susceptibility to severe disease and death (55). Interestingly, polymorphisms in CCR5, which plays important roles in leukocyte migration, have been implicated in TBE disease susceptibility and severity [reviewed in (33)]. These findings underscore the potential contribution of high cytokine expression to immunopathology and poor disease outcomes. Therefore, a better understanding of specific cytokines upregulated during acute disease could be of therapeutic value.

#### 2.3.1 NK cell responses during CNS disease

Natural killer (NK) cells are a subset of cytotoxic innate lymphocytes which play important roles in eliminating virally-infected and tumor cells. While not much is known about the role of NK cells in TBE prior to the development of CNS disease, NK cell-associated cytokines, including IL-12, IL-15, IL-18, IFN-γ, and TNF-α are upregulated in the serum of patients during severe disease (56) and NK cells can further be detected in the CSF; indicating their migration to the CNS (57). Interestingly, while NK cells detected in the peripheral blood of patients have an activated (CD57+ CD56^dim^) phenotype (56), they appear to be poorly functional, possibly indicating limited protective capacities (56). Thus, clear roles for NK cells in the context of TBE have yet to be defined, though few studies have addressed this to date. NK cell maturation and activity in cases of mild disease have not been reported and may be distinct from that observed in severe disease.

#### 2.3.2 Neutrophil responses during CNS disease

Neutrophils are important phagocytic cells during the early immune response to viral infections and are major producers of inflammatory cytokines. In tick feeding experiments, they are attracted to the bite site and can be infected with TBEV (40). However, like NK cell responses, little is understood about their role in protection prior to CNS disease. One study of TBE patients found that neutrophils are universally present in the CSF, and, that IL-8 (a neutrophil chemoattractant) is the most abundant CSF cytokine (58). In the same study, neutrophil counts were highest in the most severe cases of disease and their prolonged presence in follow-up CSF samples was associated with neurologic sequelae (58). Similarly, a study of TBE vaccine breakthrough infections found high systemic levels of IL-8 and CXCL8 (an additional neutrophil chemoattractant) during acute infection which remained elevated into convalescence (59). Supporting this, work in a mouse LGTV model demonstrated increased neutrophil migration into the CNS, and, further, that depletion of neutrophils resulted in decreased viral loads, decreased immunopathology, and improved survival (60). Together these findings indicate a role for neutrophils in immunopathology during severe TBE disease, making them a potential target for immunotherapeutic approaches.

## 3 Cellular immune responses to TBEV infection and vaccination

Cellular immune responses comprise one arm of the so-called “adaptive” immune system. As discussed, a key feature of adaptive immunity is the ability to form immune memory following primary pathogen exposure, which is able to provide rapid protective responses upon later pathogen re-encounter. Cellular immunity relies primarily on T cell-mediated immune responses. While T cell responses during TBEV infection (**Figure 1A**) or TBE vaccination (**Figure 1B**) are substantially less well-understood than humoral responses, they seem to play an important role in protection. As with early innate immune responses, a major issue in our understanding of cellular immunity during TBEV infection is that most studies are conducted in patients with relatively severe disease, and late in the disease course – namely after CNS involvement. This is critical for identifying potential areas for therapeutic development, though is not representative of the majority of TBE cases. As a consequence, our understanding of what constitutes an “ideal” protective immune response is limited.

### 3.1 CD4+ T cells

CD4+ T cells are key producers of cytokines which help to drive antiviral immune responses. They are also essential to provide the B cell help necessary for antibody production. Like other orthoflaviviruses, TBEV encodes seven non-structural proteins [NS1, NS2a, NS2b, NS3, NS4a, NS4b, and NS5 (3, 6, 28)], but has only three structural proteins: the C (capsid) protein and two membrane-associated proteins, prM/M (precursor of Membrane/Membrane) and E (envelope) (3, 6, 28). The structural proteins appear to be the major targets of CD4+ T cell responses during infection (61, 62). While they have been documented to some extent, additional information on responses to NS proteins during infection could potentially be useful in identifying additional vaccine targets, or in the development of assays which could distinguish between vaccination and infection. In clinical TBE cases, T cell activation appears to peak approximately one week after hospitalization, indicating that primary T cell responses are delayed until the CNS phase of illness, at least in cases of severe disease (63, 64). Whether this is the case in mild infections is not clear.

The majority of CD4+ T cells observed during TBEV infection are polyfunctional, producing mainly IL-2, TNF-α, and IFN-γ; the major cytokines of type 1 immune responses [(61, 64), **Figure 1A**]. IFN-γ-mediated responses, in particular, are known to be important in type 1 control of viral infections and are often also associated with direct antiviral effector functions in CD4+ T cells. CD4+ T cells appear to have a moderate activation phenotype during TBE infection, suggesting that they may play a less important role in direct viral clearance, but also, may have less immunopathogenic potential, than, for example, CD8+ T cells (65). In line with their potential protective roles, adoptive transfer of CD4+ T cells into TBEV-infected Severe Combined Immunodeficiency (SCID; no T or B cells) mice was demonstrated to protect against lethal disease (44).

Following vaccination, CD4+ T cell counts positively correlate with anti-TBEV antibody responses (66) and vaccine responders have increased antigen-specific T cell proliferation compared to non-responders (67). Like infection, vaccination elicits CD4+ T cells specific to TBEV C, prM/M, and envelope E proteins (61, 68), with cells specific to two of the four alpha helices of C and domain III of E (EDIII) dominating the response (62). TBEV-specific CD4+ T cells generated by vaccination, however, appear to react to a narrower range of viral targets compared to those generated by infection (61, 62) and while the majority of CD4+ T cells generated by vaccination, like infection, are polyfunctional (61, 64), vaccination-elicited IFN-γ responses reach only about half those elicited by infection in terms of both magnitude and number of virus-derived peptides capable of eliciting IFN-γ responses ((61), **Figure 1 B**). Vaccine responses further tend to be biased towards IL-2 and TNF-α production compared to infection [(64), **Figure 1B**]. Ideally, vaccines would elicit more robust IFN-γ-producing CD4+ T cell responses. However, whether CD4+ T cells are indeed directly involved protection from infection (including asymptomatic or mild and severe infection) as has been suggested in animal studies (44) is not yet clear, but would be valuable to understand.

### 3.2 CD8+ T cells

CD8+ T cells, also known as cytotoxic T cells, play crucial roles in viral infection by identifying and destroying infected host cells, thereby limiting the spread of the virus in the body. During TBEV infection, it appears that NS proteins are important targets of the CD8+ T cell response; among 6 CD8+ T cell epitopes identified in one study, all were derived from viral nonstructural (NS) proteins (69). This is in contrast to CD4+ T cells, which appear to target structural proteins, demonstrating differences in viral targets between T cell types. In TBE patients, at the peak of the T cell response 1 week following hospitalization, CD8+ T cell activation was substantially increased compared to CD4+ T cells, indicating that responses tend to be CD8-dominated (65). These CD8+ T cells further displayed an effector phenotype (CD45RA-CCR7) (65, 69), and had a highly-activated Eomes+Ki67+T-bet+ transcriptional profile (65). These effectors, however, tended to be monofunctional (65). Following acute infection, as patients became convalescent, antigen-specific CD8+ T cells transitioned to an Eomes-Ki67-T-bet+ phenotype (65), consistent with a type 1 effector memory (TEM) population. Interestingly, in comparing Human Leukocyte Antigen (HLA)-A2- and HLA-B7-restricted CD8+ cells, the most prevalent phenotype among HLA-A2-restricted cells was effector memory (TEM), whereas the HLA-B7-restricted population was predominantly of a TEM-reexpressing CD45RA (TEMRA) phenotype (69), indicating that CD8+ T cells with distinct viral specificities may have different memory phenotypes.

While the immune responses to acute CNS disease is CD8-dominated (**Figure 1A**), the role of these CD8+ T cells in immunopathology versus protection during TBE disease is not clear. That nearly all studies assessing CD8+ T cell responses utilize patients with severe disease limits our understanding of whether this population is an important mediator of protection in mild or asymptomatic illness. Results in animal studies have also been mixed. CCR5-deficient animals experienced a temporal lag in lymphocyte migration into the CNS which led to increased mortality in LGTV infection, which could be alleviated by adoptive transfer of wildtype (but not CCR5-deficient) T cells, demonstrating the importance to T cell responses in protection from lethal infection (60). In contrast, survival following lethal TBEV infection in SCID and CD8-knockout mice was increased compared to wildtype or mice with adoptively transferred CD8+ T cells, demonstrating that CD8+ T cells can also contribute to lethal infection (44). Similarly, CD8+ T cell infiltrates are commonly found in the post-mortem brains of fatal TBE cases (70–72), and a separate study found that, in severely infected patients, nearly all virus-specific CD8+ T cells expressed α4 and β1 integrins (VLA-4), which are important in lymphocyte homing and the ability of cells to cross the blood-brain barrier (69). However, breakdown of the BBB during infection in mice was observed in both wildtype and CD8-knockout animals, indicating that CD8+ T cells are not responsible for BBB permeability during disease (46). Interestingly, in a mouse model of TBEV infection, TCR CDR3 gene usage differed between lethally and non-lethally infected mice, although no differences in T-cell activation markers or apoptosis-related genes were observed, suggesting that disease severity may be related to antigen specificity, rather than simply the number or activation level of brain-infiltrating T cells (73). While the mechanism by which TBEV causes CNS destruction remains unclear, it may involve a combination of both direct neuronal damage by the virus and indirect damage caused by the immune response.

In contrast to infection, data on CD8+ T cell responses following vaccination are limited (**Figure 1B**). While T cell receptor (TCR) sequencing analysis has demonstrated that CD8+ T cells do respond and expand following vaccination (74), few TBEV-reactive CD8+ T cells are detectable in the peripheral blood of vaccinees and overall vaccine responses are clearly CD4-biased (68), suggesting that CD8+ T cells play a minor role in vaccine-elicited protection. As mentioned, the primary CD8+ T cell targets during infection are viral NS proteins (69). Importantly, these proteins are expressed during active viral replication and, therefore, are detectable in only small quantities in currently used inactivated vaccines (75). This may also explain, in part, the low CD8+ T cell responses to vaccination. While TBEV infection is thought to elicit lifelong protective CD8+ responses, little information on this is available (76, 77). Differences in the epitopes targeted by infection versus vaccination could potentially play a role. However, as CD8+ T cell responses likely contribute to both protection and immunopathology, it is unclear whether vaccines which promote strong CD8+ T cell responses would be desirable. Their powerful anti-viral functions could provide rapid protection, though, if appropriately harnessed. While not yet explored in the context of TBEV, tissue-resident immune responses, including tissue-resident memory T cells [TRM (78, 79)] could represent interesting potential targets for future study. Perhaps such skin-localized T cell immunity, elicited by a vaccine, for example, could help to provide rapid protection against the development of disease at the site of initial infection and prior to viral spread.

## 4 Humoral immune responses in TBEV infection and vaccination

Humoral immunity, mediated by antibodies produced by B cells, is the arm of the adaptive immune response that functions to neutralize and eliminate extracellular microbes and microbial toxins. It plays a vital role in protection from viral infections with antibodies functioning to neutralize virus binding and entry to host cells, as well as coating viral particles to induce their uptake and destruction by phagocytic immune cells. The long-term maintenance of memory B cells further enables the immune system to mount faster and more effective responses upon reinfection as these cells quickly differentiate into antibody-producing plasma cells when they encounter the same virus again, helping to eliminate the virus before it can cause widespread infection and disease. Humoral immunity is thought to play a crucial role in protection against TBE by generating antibodies that specifically target TBEV. These antibodies neutralize the virus and prevent its spread, helping to limit the severity of infection and providing long-term immunity against future TBEV exposures.

### 4.1 B cells

In contrast to T cell responses, which, as discussed, peak approximately 1 week post-symptomatic CNS disease, TBEV-specific humoral responses are observed earlier in infection. Among TBE patients, antibody-secreting cells, activated B cells which have begun to produce antibodies, are already detected at the time of hospital admission and do not appear to expand further, indicating that they likely develop prior to CNS-symptomatic infection (80). Similarly, in the same study, all patients presented with detectable TBEV-specific IgM and IgG upon admission which were maintained into convalescence (80). In comparing immune responses detectable in the peripheral blood and CNS during TBEV infection, several studies have suggested that type 1 cellular immune responses tend to be higher in the CSF (49, 51, 57, 81), while Th17-type (dominated by follicular helper T cells which provide help to antibody-producing B cells) and B cell responses tend to be more pronounced in the blood (49, 51, 57, 81). Together, these findings are consistent with the idea that B cells and subsequent antibody-mediated responses are important in controlling the viremic stages of infection where TBEV may spread and seed several peripheral tissues (**Figure 1A**).

The E protein is comprised of three domains (EDI, EDII, and EDIII) and a C-terminal stem-anchor region (82). In TBE-vaccinated individuals, anti-EDIII memory B cell clones are expanded, consistent with the important role of the EDIII in viral infection (83). Neutralizing antibody responses, however, are reduced compared to those observed in infected individuals (83). Age appears to influence the functionality of memory B cell populations established in response to vaccination with individuals aged 60+ generating approximately 3-fold fewer virus-specific memory B cells compared to younger individuals aged 20-30 (66). Consistent with this, virus-specific IL-2-producing CD4+ T cell responses were reduced among older individuals, suggesting that decreased antibody responses in the elderly are likely due to a combination of reduced B cell and CD4+ T cell responses (66). After booster vaccination, similar frequencies of “reactivated” B cells were observed in both groups, but overall antibody production remained lower in older individuals, suggesting reduced functionality (66). It is clear, however, that memory responses to TBEV can be maintained for long periods of time (perhaps decades) at low levels in the body (**Figure 1B**), including into older age. This is particularly evident in the context of TBE vaccination where, even in individuals a decade or more post-last vaccination that have become seronegative, booster vaccination results in anamnestic responses reaching levels considered to be seroprotective (84, 85). That antibody responses can rapidly recalled upon booster vaccination indicates the important role of memory B cells in protection and perhaps suggests that a subset of neutralizing antibody-producing memory B cells could be a correlate of protection. However, the memory B cell populations driving these responses are not well-described nor understood among vaccinated or TBEV-infected individuals. The nature of B cell memory established following TBE vaccination, including their specificities and protective capacities, remains an important area for further research.

### 4.2 Antibody responses

The dynamics of antibody responses following TBEV infection and primary vaccination have been well-reviewed (7, 10, 86–88) and are generally better understood than cellular immune responses. IgM antibodies are observed early during symptomatic disease, whereas IgG antibodies peak later into convalescence (89). At the time of the first CNS symptoms, TBEV-specific IgM is present in serum; within the first six days, IgM levels rise and decreases again by six weeks, but remain detectable for several months after infection (90, 91). Serum IgG levels increase only moderately during the CNS phase of the infection, peaking approximately 6 weeks after the onset of the first neurological symptoms; however, their presence is long-lasting (86, 90–92). After infection, IgG can persist life-long and is thought to play a key role in preventing reinfection (91, 93).

While T cells target a variety of TBEV proteins, B cell and antibody-mediated responses seem to primarily target E and, to some extent, NS1. The E glycoprotein mediates viral binding and entry into host cells [heparan sulfate has been identified as a likely host cell receptor for TBEV (94)] and is the primary target for neutralizing antibodies both during infection and in response to vaccination (95). More than 12 distinct epitopes have been identified which elicit antibodies characterized by varying degrees of neutralization potency (95). Antibodies specific for NS proteins do not directly neutralize virus infectivity, but may protect via other effector mechanisms (95). Compared to whole-virus antibodies, anti-NS1 antibodies are produced at lower titers and appear later during disease (96, 97). Several studies have shown, though, that NS1-specific antibodies play a protective role against TBE (75, 98–103) and detection of anti-NS1 antibodies may distinguish infection from vaccination, as non-structural proteins are produced mainly during viral replication (97, 104, 105). Recent research has shown, however, that NS1-specific antibodies can be generated by vaccination, although the titers in vaccinees remain low compared to TBE patients, making it unlikely that vaccination-induced anti-NS1 antibodies play a significant role in protection (20).

Here we focus on two specific aspects of the antibody response to TBEV infection and vaccination; neutralization potential, and intrathecal antibodies.

#### 4.2.1 Neutralization potential

Neutralizing antibodies are thought to be key players in the protective immune response generated following TBE vaccination, and, indeed, they are considered by the WHO as a surrogate measurement for the “correlate of protection” against disease (106), with titers of 1:10 or greater generally considered as sufficient evidence of protection (107). Orthoflavivirus neutralization is a “multiple hit” phenomenon requiring engagement by more than a single antibody (95). Epitopes have been mapped to sites within each of the three E protein domains, to domain-overlapping sites within the same protein monomer, to E protein dimer-specific sites involving residues from both monomers, and to sites not represented by soluble forms of the E protein but requiring the quaternary arrangement in virus particles (108). Potent orthoflaviviral neutralizing antibodies have been shown to interfere with the process of virus-induced membrane fusion (83, 109, 110). Other antibodies are thought to block the binding of the virion to cellular receptors, block the interaction of the virion with cellular receptors through steric hindrance, or block membrane fusion inside endosomes or phagosomes through the cross-linking of E molecules (111). It is plausible that the mechanism of neutralization of many E-specific antibodies involves both steps of virus entry and is modulated by the composition of antibody populations in polyclonal sera (108), complicating potential therapeutic development.

The dominance of antibodies to different E domains is strongly affected by both host-species-specific and virus-specific factors. Many of the most potent orthoflaviviral neutralizing antibodies characterized to date recognize the upper lateral surface of EDIII that protrudes from the surface of the virion; these antibodies contribute strongly to the neutralizing response in mice but not in humans (95, 112). Antibodies against EDI and EDII are dominant in the human immune response against TBEV (113). However, binding of some EDIII- and EDII-specific antibodies could result in rearrangement of the surface of glycoprotein E and increase the availability of the fusion loop to specific antibodies (114, 115). Due to the potent neutralizing activity of anti-EDIII antibodies, a vaccine strategy focusing on this domain could potentially be beneficial (83).

It is known that the specificity of antibodies produced upon infection and vaccination differ and anti-TBEV neutralizing antibody titers are much higher among infected, compared to vaccinated individuals (76, 116). Since most TBE vaccines are inactivated virus preparations [reviewed in (7–10)], the amount of antigen available to the immune system is fixed and responses are biased towards CD4+ T cells by virtue of the exogenous nature of the antigens (CD4+ and CD8+ T cells respond to exogenously- and endogenously-derived antigens, respectively). By contrast, infection allows for a larger and more persistent supply of antigen due to viral replication and active infection of host cells additionally drives CD8+ T cell responses. These distinct responses to infection and vaccination likely explain the substantially higher neutralization titers associated with infection (65, 117), as well as the CD4+ T cell-biased response to vaccination. On the other hand, the development of neutralizing antibodies in the acute phase of disease is delayed compared with their rapid appearance following vaccination (76, 116). Interestingly, the functional activity of antibodies appears to be individually imprinted; for vaccinated individuals, there is a tendency to maintain a specific antibody profile established during initial priming of the immune response (118).

Infection with one orthoflavivirus results in the production of both species-specific and cross-reactive antibodies due to the high antigenic similarity among various orthoflaviviruses (119). Such orthoflavivirus cross-neutralizing antibodies can be induced during the acute phase of infection and disease (83, 120–122). They are, however, not typically durable and cross-neutralization is thought to be retained only a few months (123). Furthermore, while cross-neutralization may offer some level of cross-protection, pre-existing immunity to other orthoflaviviruses can also hinder and alter the immune response to TBEV vaccination (124, 125). While TBE vaccination does not appear to offer protection against other orthoflaviviral infections, it is generally believed that TBEV vaccines can protect from infection by both homologous and heterologous TBEV subtypes (122, 126–128). However, some studies of European vaccines have demonstrated reduced protection against some Far Eastern and Siberian subtype strains (127, 129). Thus, the question whether or not vaccines sufficiently protect against heterologous strains warrants further investigation, optimally including viral strains other than the prototypes of each subtype.

Due to their potential for protection, antiviral antibodies may be valuable as therapeutics. Indeed, several studies have evaluated the use of monoclonal antibodies, chimeric antibodies, or intravenous immunoglobulin for TBE therapy. Concern over reports of antibody-dependent enhancement (ADE) after post-exposure prophylaxis in children, however, have led to discontinuation of the use of anti-TBEV immunoglobulins in Europe. While antibodies have been shown to be protective when given before, and even after, infection (54, 83, 130–133) there remains, to date, no consensus on whether it is safe to use antibody therapy as post-exposure prophylaxis against TBEV. Thus, in addition to the use of specific and non-specific immunoglobulins, the administration of recombinant antibodies may be potential approach to immunotherapy (7, 134).

#### 4.2.2 Intrathecal antibodies

While circulating antibody responses in the serum are well-described and several studies have demonstrated protective roles for early serologic response in the blood (135–138), roles of antibody responses in the CNS (intrathecal antibodies, within the CSF) during infection are less understood. IgM is produced locally within the CNS but is not passively transferred into the CSF to a great extent, indicating that TBEV-specific antibody-secreting cells or plasma cells must have first entered the CNS. At the onset of symptoms, IgM in the CSF can be found in only up to 50% of patients (91, 137, 139), in contrast to their detection in the blood in nearly all patients at this timepoint. However, within 10 days after onset of illness, CSF IgM is almost invariably detectable and peak concentrations are reached approximately 14 days after CNS symptom onset (24, 91, 140, 141). IgG, which naturally follows a slower kinetic compared to IgM, increases only moderately during acute CNS disease, but peaks in CSF approximately 6 weeks after the first neurological disease symptoms, well into convalescence (86, 90, 91). At the timepoint of hospitalization or within one month, IgG is detectable in the CSF in 43% or 92% of patients, respectively (137, 139). Unlike IgM, however, IgG is transferred passively to the CSF, especially during inflammatory processes in the CNS that disturb the blood-brain barrier.

Intrathecal synthesis of total IgG, IgM, and also IgA appears to be higher in severe, compared to mild, disease (24, 137). On the other hand, a lower IgG intrathecal index at hospital admission is a possible risk factor for developing persistent sequelae (142), and the intrathecal anti-TBEV IgM response may be associated with significantly quicker resolution of the cellular CSF infiltrate (137). Thus, the role of intrathecally produced antibodies remains somewhat unclear and would benefit from further investigations.

## 5 Durability of protection

Following infection antibody titers remains stable at high levels over many years (76, 77). Furthermore, and in contrast to vaccination, titers following infection are comparable between both older and younger individuals. While it is thought that IgG generated following infection persist lifelong and may mediate protection from reinfection (86), a comparison of seroprevalence and average TBE incidence rates from the 1980s through 2001 suggests that previous infection actually may not induce lifelong immunity (143). Thus, it remains to be determined whether TBE mediates lasting protection against TBEV reinfection.

In contrast to infection, seropersistence data after primary and booster vaccinations with both European vaccines (77, 84, 85, 124, 144–161) demonstrates that TBEV-neutralizing antibody titers induced by vaccination decline over time (144, 145) but persist between 5 to 10 years at least (145–150). Lasting protection against TBE is maintained by booster vaccinations. Manufacturers’ recommendations for both European TBE vaccines include a first booster three years after completion of the three-dose primary vaccination series. The need for a first, three-year booster is not completely clear. In one study, seropositivity among 18-50 year-olds declined to 89-92% after two to three years (144) and another study found that only 51% of individuals without a first booster had protective titers eight or more years later (162). Studies evaluating the persistence of anti-TBEV antibodies following primary immunization have demonstrated that titers decline at a slower rate after at least one booster and that protective titers can be subsequently maintained up to 10 years or more (85, 144, 149, 150, 156, 158, 162–167).

Additionally, and in contrast to infection where titers remain high in older individuals at levels comparable to those observed in younger individuals, the magnitude of antibody responses following TBE booster immunization is reduced among adults aged 50+ (84, 145, 148, 155, 158, 165), as is the duration of seropositivity (85, 148, 164, 165). While all ages are at risk for TBE, those aged 50+ make up the majority of cases and have the greatest incidence of severe disease (168, 169). Vaccine responses, however, are reduced in this age group. Rates of antibody seroconversion are lower (144), titers are reduced (85, 145, 148), and long-term seropositivity is reduced (85, 150). Rates of vaccination failure are also higher (170). Work from Sweden has demonstrated reduced vaccination failures among older individuals with additional booster vaccine doses (171), as well as increased titers among those 50+ randomized to receive a four-dose primary vaccination schedule (172). Taken together, these findings suggest that the length of booster intervals should be carefully considered in light of age dependent differences in antibody durability.

While antibody responses are typically used to assess responsiveness to TBE vaccination, field effectiveness data (173–177) indicate that antibody responses may not necessarily be a suitable surrogate for vaccine effectiveness (VE) estimates. Field effectiveness data from several studies indicates that VE for European TBE vaccines ranges from 90-99% [(173–175, 178–180) reviewed in (9, 181, 182)]. Similarly, studies throughout Europe have estimated the frequency of TBE vaccination failures at approximately 2-7% (92, 169–171, 183–185). Furthermore, increasing evidence shows that TBE VE remains high (>90%) for at least 10 years after completion of the primary series (175–177), despite the clear decline in both total IgG and neutralizing antibody titers over time, indicating that antibody responses do not always clearly track with, and may underestimate, protection. This, in turn, suggests important roles for other immune populations in maintaining long-term protection. While memory B cells, for example, have been shown to ensure anamnestic antibody responses even after extended periods post-vaccination and even in individuals who have become seronegative (84, 85), there remains a need for future research investigating the sustained responsiveness of CD4+ and/or CD8+ T cells after infection and vaccination.

## 6 Discussion

Here we discuss the immune responses to TBEV infection and TBE vaccination, outlining points where “correlates of protection” might play key roles, and highlighting outstanding questions (**Table 1**). During the early stages of infection, for example, the immune response is critically shaped by local responses within the skin. Whether there might be roles for local trained innate immune responses or “tissue-resident” T or B cell populations in protection in previously exposed hosts or following vaccination remain interesting areas worth further exploration, potentially allowing for rapid protection at the initial infection site before viral spread. Similarly, as cytokine expression patterns could contribute to either protection, or immunopathology, a better understanding of specific cytokines upregulated early on in acute TBE disease, or after vaccination, could have therapeutic value or provide insights into differences between vaccine responders and low- or non-responders.

**Table 1:** Summary of outstanding questions discussed in this review.

Immune subset	Outstanding questions
**Skin-resident immune populations**	• As the host must develop immune responses protective in distinct environments following infection, it is appropriate to consider site-specific immunity; as initial viral replication occurs in the skin, skin-resident immune populations could represent an interesting area of future study with implications for vaccination.
**Cytokines**	• Cytokine responses are responsible for cellular trafficking, including trafficking of leucocytes into the central nervous system, during TBE, and represent potential therapeutic targets. • Further study of cytokine responses following vaccination could potentially provide insights into differences between vaccine responders and low/non-responders.
**NK cells and neutrophiles**	• While neutrophils are a major player in the immune response to infection, they appear to have an immunopathologic role in severe infections, making them a potential target for immunotherapeutic approaches.
**CD4+ T cells**	• CD4+ T cells are generated by both infection and vaccination, though their functional capacities differ. Ideally, vaccines would elicit more robust IFN-γ-producing CD4+ T cell responses. • Whether CD4+ T cells are directly involved in protection is not clear, but would be valuable to understand from the perspective of vaccine development. • While CD4+ responses to TBEV structural proteins are well-documented in response to vaccination and infection, additional information on responses to NS proteins during infection would be useful to identify additional vaccine targets.
**CD8+ T cells**	• As CD8+ T cells are poorly-elicited by vaccination, their responses in this context are not well-studied. As CD8+ T cells contribute to protection and immunopathology, it is unclear whether vaccines which elicit CD8+ T cell responses would be desirable. • Nearly all studies assess CD8+ T cell responses in the context of patients with severe disease, hampering understanding of whether this population is an important mediator of protection in mild or asymptomatic illness.
**Antibody response**	• The significance of the intrathecal synthesis of the specific antibodies is unclear and would benefit from further investigations. • While it is generally believed that infection mediates life-long immunity, whether this is indeed the case and by which immune subsets may warrant further investigation. • Whether vaccines sufficiently protect against heterologous strains warrants further investigation, optimally including strains other than the subtype “prototype” strains.
**B cells**	• The memory B cell populations driving the rapid recall of antibody responses upon secondary antigen contact are not well-described nor understood among vaccinated or TBEV-infected individuals. The nature of B cell memory established following TBE vaccination, including their specificities and protective capacities, remains an important area for further research.

In terms of adaptive immunity, while much work has focused on antibody responses in TBE disease and following vaccination, memory B and T cell responses also appear to act as important mediators of protection. The rapid recall of antibody (including neutralizing antibody) responses upon booster vaccination underscores the vital role played by memory B cells. Importantly, memory B cells depend heavily on initial CD4+ T cell help and it remains to be explored whether CD4+ T cells may themselves, play a role in direct viral clearance, similar to CD8+ T cells. There is, however, a key need for additional studies fousing on the functions of these adaptive immune subsets particularly in asymptomatic and mild disease, which represent “ideal” protective immune responses and could provide a baseline for what vaccine-mediated immunity “should” look like.

Importantly, monitoring the duration of immunity is also key for ensuring long-term protection, as well as for developing effective immunization strategies. Whether infection mediates life-long immunity and by which immune subsets warrants additional investigation. Similarly, as vaccine effectiveness data indicate that neutralizing antibody titers do not clearly track with protection, further understanding of which immune subsets do will be necessary for establishing reliable correlates of protection following vaccination.

## Author contributions

RA-G: Writing – original draft, Writing – review & editing, Conceptualization, Funding acquisition. PL: Writing – review & editing, Conceptualization. KZ: Conceptualization, Writing – review & editing, Funding acquisition, Writing – original draft.

## References

[1] Chiffi G Grandgirard D Leib SL Chrdle A Růžek D. Tick-borne encephalitis: A comprehensive review of the epidemiology, virology, and clinical picture. Rev Med Virol (2023) 33(5):e2470. doi: 10.1002/rmv.2470 37392370

[2] Kunze M Banović P Bogovič P Briciu V Čivljak R Dobler G. Recommendations to improve tick-borne encephalitis surveillance and vaccine uptake in europe. Microorganisms (2022) 10(7):1283. doi: 10.3390/microorganisms10071283 35889002 PMC9322045

[3] Lindquist L Vapalahti O. Tick-borne encephalitis. Lancet (2008) 371(9627):1861–71. doi: 10.1016/S0140-6736(08)60800-4 18514730

[4] Demina TV Dzhioev YP Verkhozina MM Kozlova IV Tkachev SE Plyusnin A. Genotyping and characterization of the geographical distribution of tick-borne encephalitis virus variants with a set of molecular probes. J Med Virol (2010) 82(6):965–76. doi: 10.1002/jmv.21765 20419810

[5] Dai X Shang G Lu S Yang J Xu J. A new subtype of eastern tick-borne encephalitis virus discovered in Qinghai-Tibet Plateau, China. Emerg Microbes Infect (2018) 7(1):74. doi: 10.1038/s41426-018-0081-6 29691370 PMC5915441

[6] Gritsun TS Lashkevich VA Gould EA. Tick-borne encephalitis. Antiviral Res (2003) 57(1-2):129–46. doi: 10.1016/S0166-3542(02)00206-1 12615309

[7] Ruzek D Avšič Županc T Borde J Chrdle A Eyer L Karganova G. Tick-borne encephalitis in Europe and Russia: Review of pathogenesis, clinical features, therapy, and vaccines. Antiviral Res (2019) 164:23–51. doi: 10.1016/j.antiviral.2019.01.014 30710567

[8] Kubinski M Beicht J Gerlach T Volz A Sutter G Rimmelzwaan GF. Tick-borne encephalitis virus: A quest for better vaccines against a virus on the rise. Vaccines (Basel) (2020) 8(3). doi: 10.3390/vaccines8030451 PMC7564546 32806696

[9] Steffen R Erber W Schmitt HJ. Can the booster interval for the tick-borne encephalitis (TBE) vaccine ‘FSME-IMMUN’ be prolonged? - A systematic review. Ticks Tick Borne Dis (2021) 12(5):101779. doi: 10.1016/j.ttbdis.2021.101779 34298356

[10] Rampa JE Askling HH Lang P Zens KD Gültekin N Stanga Z. Immunogenicity and safety of the tick-borne encephalitis vaccination (2009-2019): A systematic review. Travel Med Infect Dis (2020) 37:101876. doi: 10.1016/j.tmaid.2020.101876 32931931

[11] Lipowski D Popiel M Perlejewski K Nakamura S Bukowska-Osko I Rzadkiewicz E. A cluster of fatal tick-borne encephalitis virus infection in organ transplant setting. J Infect Dis (2017) 215(6):896–901. doi: 10.1093/infdis/jix040 28453842

[12] Wahlberg P Saikku P Brummer-Korvenkontio M. Tick-borne viral encephalitis in Finland. The clinical features of Kumlinge disease during 1959–1987. J Internal Med (1989) 225(3):173–7. doi: 10.1111/j.1365-2796.1989.tb00059.x 2703799

[13] Morozova OV Panov VV Bakhvalova VN. Innate and adaptive immunity in wild rodents spontaneously and experimentally infected with the tick-borne encephalitis virus. Infect Genet Evol (2020) 80:104187. doi: 10.1016/j.meegid.2020.104187 31927073

[14] Labuda M Jones LD Williams T Nuttall PA. Enhancement of tick-borne encephalitis virus transmission by tick salivary gland extracts. Med Vet Entomol (1993) 7(2):193–6. doi: 10.1111/j.1365-2915.1993.tb00674.x 8481537

[15] Nuttall PA. Tick saliva and its role in pathogen transmission. Wiener klinische Wochenschrift (2019) 135(7):165–76. doi: 10.1007/s00508-019-1500-y PMC10118219 31062185

[16] Kotál J Langhansová H Lieskovská J Andersen JF Francischetti IMB Chavakis T. Modulation of host immunity by tick saliva. J Proteomics (2015) 128:58–68. doi: 10.1016/j.jprot.2015.07.005 26189360 PMC4619117

[17] Kaiser R. Tick-borne encephalitis. Infect Dis Clin North Am (2008) 22(3):561–75, x. doi: 10.1016/j.idc.2008.03.013 18755391

[18] Kaiser R. [Tick-borne encephalitis]. Nervenarzt (2016) 87(6):667–80. doi: 10.1007/s00115-016-0134-9 27225401

[19] Bogovic P Strle F. Tick-borne encephalitis: A review of epidemiology, clinical characteristics, and management. World J Clin Cases (2015) 3(5):430–41. 10.12998/wjcc.v3.i5.430 10.12998/wjcc.v3.i5.430 PMC4419106 25984517

[20] Ackermann-Gäumann R Brêchet A Smetana J Salát J Lienhard R Croxatto A. Vaccination against tick-borne encephalitis elicits a detectable NS1 IgG antibody response. J Virol Methods (2023) 322:114831. doi: 10.1016/j.jviromet.2023.114831 37838083

[21] BAG. Zahlen zu Infektionskrankheiten Zeckenenzephalitis FSME: Swiss Federal Office of Public Health. Available at: https://www.bag.admin.ch/bag/de/home/zahlen-und-statistiken/zahlen-zu-infektionskrankheiten.exturl.html/aHR0cHM6Ly9tZWxkZXN5c3RlbWUuYmFnYXBwcy5jaC9pbmZyZX/BvcnRpbmcvZGF0ZW5kZXRhaWxzL2QvZnNtZS5odG1sP3dlYmdy/YWI9aWdub3Jl.html.

[22] Haglund M Günther G. Tick-borne encephalitis–pathogenesis, clinical course and long-term follow-up. Vaccine (2003) 21 Suppl 1:S11–8. doi: 10.1016/S0264-410X(02)00811-3 12628810

[23] Kaiser R. [Epidemiology and progress of early summer meningoencephalitis in Baden-Württemberg between 1994 and 1999. A prospective study of 731 patients]. Dtsch Med Wochenschr (2000) 125(39):1147–53. doi: 10.1055/s-2000-7668 11075241

[24] Kaiser R Holzmann H. Laboratory findings in tick-borne encephalitis–correlation with clinical outcome. Infection (2000) 28(2):78–84. doi: 10.1007/s150100050051 10782392

[25] Kaiser R. Tick-borne encephalitis: Clinical findings and prognosis in adults. Wien Med Wochenschr (2012) 162(11-12):239–43. doi: 10.1007/s10354-012-0105-0 22695809

[26] Logar M Bogovic P Cerar D Avsic-Zupanc T Strle F. Tick-borne encephalitis in Slovenia from 2000 to 2004: comparison of the course in adult and elderly patients. Wien Klin Wochenschr (2006) 118(21-22):702–7. doi: 10.1007/s00508-006-0699-6 17160611

[27] Steffen R. Tick-borne encephalitis (TBE) in children in Europe: Epidemiology, clinical outcome and comparison of vaccination recommendations. Ticks Tick Borne Dis (2019) 10(1):100–10. doi: 10.1016/j.ttbdis.2018.08.003 30241699

[28] Simmonds P Becher P Bukh J Gould EA Meyers G Monath T. ICTV virus taxonomy profile: flaviviridae. J Gen Virol (2017) 98(1):2–3. doi: 10.1099/jgv.0.000672 28218572 PMC5370391

[29] Pan Y Cai W Cheng A Wang M Yin Z Jia R. Flaviviruses: innate immunity, inflammasome activation, inflammatory cell death, and cytokines. Front Immunol (2022) 13:829433. doi: 10.3389/fimmu.2022.829433 35154151 PMC8835115

[30] Thangamani S Hermance ME Santos RI Slovak M Heinze D Widen SG. Transcriptional Immunoprofiling at the Tick-Virus-Host Interface during Early Stages of Tick-Borne Encephalitis Virus Transmission. Front Cell Infect Microbiol (2017) 7:494. doi: 10.3389/fcimb.2017.00494 29250492 PMC5716978

[31] Zheng Z Yang J Jiang X Liu Y Zhang X Li M. Tick-borne encephalitis virus nonstructural protein NS5 induces RANTES expression dependent on the RNA-dependent RNA polymerase activity. J Immunol (2018) 201(1):53–68. doi: 10.4049/jimmunol.1701507 29760190

[32] Hermance ME Santos RI Kelly BC Valbuena G Thangamani S. Immune Cell Targets of Infection at the Tick-Skin Interface during Powassan Virus Transmission. PloS One (2016) 11(5):e0155889. doi: 10.1371/journal.pone.0155889 27203436 PMC4874601

[33] Ellwanger JH Chies JAB. Host immunogenetics in tick-borne encephalitis virus infection—The CCR5 crossroad. Ticks Tick-borne Dis (2019) 10(4):729–41. doi: 10.1016/j.ttbdis.2019.03.005 30879988

[34] Kurhade C Zegenhagen L Weber E Nair S Michaelsen-Preusse K Spanier J. Type I Interferon response in olfactory bulb, the site of tick-borne flavivirus accumulation, is primarily regulated by IPS-1. J Neuroinflamm (2016) 13:22. doi: 10.1186/s12974-016-0487-9 PMC4730761 26819220

[35] Overby AK Popov VL Niedrig M Weber F. Tick-borne encephalitis virus delays interferon induction and hides its double-stranded RNA in intracellular membrane vesicles. J Virol (2010) 84(17):8470–83. doi: 10.1128/JVI.00176-10 PMC2919015 20554782

[36] Barkhash AV Perelygin AA Babenko VN Myasnikova NG Pilipenko PI Romaschenko AG. Variability in the 2′–5′-oligoadenylate synthetase gene cluster is associated with human predisposition to tick-borne encephalitis virus-induced disease. J Infect Dis (2010) 202(12):1813–8. doi: 10.1086/657418 21050126

[37] Robertson SJ Lubick KJ Freedman BA Carmody AB Best SM. Tick-borne flaviviruses antagonize both IRF-1 and type I IFN signaling to inhibit dendritic cell function. J Immunol (2014) 192(6):2744–55. doi: 10.4049/jimmunol.1302110 PMC4010128 24532583

[38] Shevtsova AS Motuzova OV Kuragina VM Akhmatova NK Gmyl LV Kondrat’eva YI. Lethal experimental tick-borne encephalitis infection: influence of two strains with similar virulence on the immune response. Front Microbiol (2016) 7:2172. doi: 10.3389/fmicb.2016.02172 28163697 PMC5247635

[39] Goonawardane N Upstone L Harris M Jones IM. Identification of host factors differentially induced by clinically diverse strains of tick-borne encephalitis virus. J Virol (2022) 96(18):e00818–22. doi: 10.1128/jvi.00818-22 PMC9517736 36098513

[40] Labuda M Austyn JM Zuffova E Kozuch O Fuchsberger N Lysy J. Importance of localized skin infection in tick-borne encephalitis virus transmission. Virology (1996) 219(2):357–66. doi: 10.1006/viro.1996.0261 8638401

[41] Saksida A Duh D Lotrič-Furlan S Strle F Petrovec M Avšič-Županc T. The importance of tick-borne encephalitis virus RNA detection for early differential diagnosis of tick-borne encephalitis. J Clin Virol (2005) 33(4):331–5. doi: 10.1016/j.jcv.2004.07.014 15919235

[42] Saksida A Jakopin N Jelovšek M Knap N Fajs L Lusa L. Virus RNA load in patients with tick-borne encephalitis, Slovenia. Emerg Infect Dis J (2018) 24(7):1315. doi: 10.3201/eid2407.180059 PMC6038823 29912706

[43] Weber E Finsterbusch K Lindquist R Nair S Lienenklaus S Gekara NO. Type I interferon protects mice from fatal neurotropic infection with Langat virus by systemic and local antiviral responses. J Virol (2014) 88(21):12202–12. doi: 10.1128/JVI.01215-14 PMC4248936 25122777

[44] Růzek D Salát J Palus M Gritsun TS Gould EA Dyková I. CD8+ T-cells mediate immunopathology in tick-borne encephalitis. Virology (2009) 384(1):1–6. doi: 10.1016/j.virol.2008.11.023 19070884

[45] Palus M Vancova M Sirmarova J Elsterova J Perner J Ruzek D. Tick-borne encephalitis virus infects human brain microvascular endothelial cells without compromising blood-brain barrier integrity. Virology (2017) 507:110–22. doi: 10.1016/j.virol.2017.04.012 28432926

[46] Růžek D Salát J Singh SK Kopecký J. Breakdown of the blood-brain barrier during tick-borne encephalitis in mice is not dependent on CD8+ T-cells. PloS One (2011) 6(5):e20472. doi: 10.1371/journal.pone.0020472 21629771 PMC3100324

[47] Grygorczuk S Osada J Toczyłowski K Sulik A Czupryna P Moniuszko-Malinowska A. The lymphocyte populations and their migration into the central nervous system in tick-borne encephalitis. Ticks Tick Borne Dis (2020) 11(5):101467. doi: 10.1016/j.ttbdis.2020.101467 32723646

[48] Grygorczuk S Czupryna P Pancewicz S Świerzbińska R Dunaj J Siemieniako A. The increased intrathecal expression of the monocyte-attracting chemokines CCL7 and CXCL12 in tick-borne encephalitis. J Neurovirol (2021) 27(3):452–62. doi: 10.1007/s13365-021-00975-z 33876413

[49] Toczylowski K Grygorczuk S Osada J Wojtkowska M Bojkiewicz E Wozinska-Klepadlo M. Evaluation of cerebrospinal fluid CXCL13 concentrations and lymphocyte subsets in tick-borne encephalitis. Int J Infect Dis (2020) 93:40–7. doi: 10.1016/j.ijid.2020.01.023 31978584

[50] Lepej SZ Misić-Majerus L Jeren T Rode OD Remenar A Sporec V. Chemokines CXCL10 and CXCL11 in the cerebrospinal fluid of patients with tick-borne encephalitis. Acta Neurol Scand (2007) 115(2):109–14. doi: 10.1111/j.1600-0404.2006.00726.x 17212614

[51] Bogovič P Kastrin A Lotrič-Furlan S Ogrinc K Avšič Županc T Korva M. Comparison of laboratory and immune characteristics of the initial and second phase of tick-borne encephalitis. Emerg Microbes Infect (2022) 11(1):1647–56. doi: 10.1080/22221751.2022.2086070 PMC9225760 35657098

[52] Atrasheuskaya AV Fredeking TM Ignatyev GM. Changes in immune parameters and their correction in human cases of tick-borne encephalitis. Clin Exp Immunol (2003) 131(1):148–54. doi: 10.1046/j.1365-2249.2003.02050.x PMC1808605 12519399

[53] Zidovec-Lepej S Vilibic-Cavlek T Ilic M Gorenec L Grgic I Bogdanic M. Quantification of antiviral cytokines in serum, cerebrospinal fluid and urine of patients with tick-borne encephalitis in Croatia. Vaccines (2022) 10(11):1825. doi: 10.3390/vaccines10111825 36366333 PMC9698853

[54] Pokorna Formanova P Palus M Salat J Hönig V Stefanik M Svoboda P. Changes in cytokine and chemokine profiles in mouse serum and brain, and in human neural cells, upon tick-borne encephalitis virus infection. J Neuroinflammation (2019) 16(1):205. doi: 10.1186/s12974-019-1596-z 31699097 PMC6839073

[55] Palus M Vojtíšková J Salát J Kopecký J Grubhoffer L Lipoldová M. Mice with different susceptibility to tick-borne encephalitis virus infection show selective neutralizing antibody response and inflammatory reaction in the central nervous system. J Neuroinflammation (2013) 10:77. doi: 10.1186/1742-2094-10-77 23805778 PMC3700758

[56] Blom K Braun M Pakalniene J Lunemann S Enqvist M Dailidyte L. NK cell responses to human tick-borne encephalitis virus infection. J Immunol (2016) 197(7):2762–71. doi: 10.4049/jimmunol.1600950 27543616

[57] Tomazic J Ihan A. Flow cytometric analysis of lymphocytes in cerebrospinal fluid in patients with tick-borne encephalitis. Acta Neurol Scand (1997) 95(1):29–33. doi: 10.1111/j.1600-0404.1997.tb00064.x 9048982

[58] Grygorczuk S Świerzbińska R Kondrusik M Dunaj J Czupryna P Moniuszko A. The intrathecal expression and pathogenetic role of Th17 cytokines and CXCR2-binding chemokines in tick-borne encephalitis. J Neuroinflamm (2018) 15(1):115. doi: 10.1186/s12974-018-1138-0 PMC5909263 29678185

[59] Pavletič M Korva M Knap N Bogovič P Lusa L Strle K. Upregulated intrathecal expression of VEGF-A and long lasting global upregulation of proinflammatory immune mediators in vaccine breakthrough tick-borne encephalitis. Front Cell Infect Microbiol (2021) 11:696337. doi: 10.3389/fcimb.2021.696337 34277474 PMC8281926

[60] Michlmayr D Bardina SV Rodriguez CA Pletnev AG Lim JK. Dual function of ccr5 during langat virus encephalitis: reduction in neutrophil-mediated central nervous system inflammation and increase in T cell-mediated viral clearance. J Immunol (2016) 196(11):4622–31. doi: 10.4049/jimmunol.1502452 PMC4973474 27183602

[61] Aberle JH Schwaiger J Aberle SW Stiasny K Scheinost O Kundi M. Human CD4+ T helper cell responses after tick-borne encephalitis vaccination and infection. PloS One (2015) 10(10):e0140545. doi: 10.1371/journal.pone.0140545 26465323 PMC4605778

[62] Schwaiger J Aberle JH Stiasny K Knapp B Schreiner W Fae I. Specificities of human CD4+ T cell responses to an inactivated flavivirus vaccine and infection: correlation with structure and epitope prediction. J Virol (2014) 88(14):7828–42. doi: 10.1128/JVI.00196-14 PMC4097808 24789782

[63] Blom K Cuapio A Sandberg JT Varnaite R Michaëlsson J Björkström NK. Cell-mediated immune responses and immunopathogenesis of human tick-borne encephalitis virus-infection. Front Immunol (2018) 9:2174. doi: 10.3389/fimmu.2018.02174 30319632 PMC6168641

[64] Varnaitė R Blom K Lampen MH Vene S Thunberg S Lindquist L. Magnitude and functional profile of the human CD4(+) T cell response throughout primary immunization with tick-borne encephalitis virus vaccine. J Immunol (2020) 204(4):914–22. doi: 10.4049/jimmunol.1901115 31924650

[65] Blom K Braun M Pakalniene J Dailidyte L Béziat V Lampen MH. Specificity and dynamics of effector and memory CD8 T cell responses in human tick-borne encephalitis virus infection. PloS Pathog (2015) 11(1):e1004622. doi: 10.1371/journal.ppat.1004622 25611738 PMC4303297

[66] Aberle JH Stiasny K Kundi M Heinz FX. Mechanistic insights into the impairment of memory B cells and antibody production in the elderly. Age (Dordr) (2013) 35(2):371–81. doi: 10.1007/s11357-011-9371-9 PMC3592966 22282053

[67] Garner-Spitzer E Wagner A Paulke-Korinek M Kollaritsch H Heinz FX Redlberger-Fritz M. Tick-borne encephalitis (TBE) and hepatitis B nonresponders feature different immunologic mechanisms in response to TBE and influenza vaccination with involvement of regulatory T and B cells and IL-10. J Immunol (2013) 191(5):2426–36. doi: 10.4049/jimmunol.1300293 23872054

[68] Gomez I Marx F Saurwein-Teissl M Gould EA Grubeck-Loebenstein B. Characterization of tick-borne encephalitis virus-specific human T lymphocyte responses by stimulation with structural TBEV proteins expressed in a recombinant baculovirus. Viral Immunol (2003) 16(3):407–14. doi: 10.1089/088282403322396190 14583154

[69] Lampen MH Uchtenhagen H Blom K Varnaitė R Pakalniene J Dailidyte L. Breadth and dynamics of HLA-A2- and HLA-B7-restricted CD8(+) T cell responses against nonstructural viral proteins in acute human tick-borne encephalitis virus infection. Immunohorizons (2018) 2(6):172–84. doi: 10.4049/immunohorizons.1800029 31022685

[70] Gelpi E Preusser M Laggner U Garzuly F Holzmann H Heinz FX. Inflammatory response in human tick-borne encephalitis: analysis of postmortem brain tissue. J Neurovirol (2006) 12(4):322–7. doi: 10.1080/13550280600848746 16966222

[71] Gelpi E Preusser M Garzuly F Holzmann H Heinz FX Budka H. Visualization of Central European tick-borne encephalitis infection in fatal human cases. J Neuropathol Exp Neurol (2005) 64(6):506–12. doi: 10.1093/jnen/64.6.506 15977642

[72] Sendi P Hirzel C Pfister S Ackermann-Gäumann R Grandgirard D Hewer E. Fatal outcome of european tick-borne encephalitis after vaccine failure. Front Neurol (2017) 8:119. doi: 10.3389/fneur.2017.00119 28421031 PMC5377060

[73] Fujii Y Hayasaka D Kitaura K Takasaki T Suzuki R Kurane I. T-cell clones expressing different T-cell receptors accumulate in the brains of dying and surviving mice after peripheral infection with far eastern strain of tick-borne encephalitis virus. Viral Immunol (2011) 24(4):291–302. doi: 10.1089/vim.2011.0017 21830901

[74] Sycheva A Komech E Pogorelyy M Minervina A Urazbakhtin S Salnikova M. Inactivated tick-borne encephalitis vaccine elicits several overlapping waves of T cell response. Front Immunol (2022) 13. doi: 10.3389/fimmu.2022.970285 PMC9449805 36091004

[75] Salat J Mikulasek K Larralde O Pokorna Formanova P Chrdle A Haviernik J. Tick-borne encephalitis virus vaccines contain non-structural protein 1 antigen and may elicit NS1-specific antibody responses in vaccinated individuals. Vaccines (Basel) (2020) 8(1). doi: 10.3390/vaccines8010081 PMC7157539 32059489

[76] Remoli ME Marchi A Fortuna C Benedetti E Minelli G Fiorentini C. Anti-tick-borne encephalitis (TBE) virus neutralizing antibodies dynamics in natural infections versus vaccination. Pathog Dis (2015) 73(2):1–3. doi: 10.1093/femspd/ftu002 25722483

[77] Baldovin T Mel R Bertoncello C Carpenè G Soppelsa F Giliberti A. Persistence of immunity to tick-borne encephalitis after vaccination and natural infection. J Med Virol (2012) 84(8):1274–8. doi: 10.1002/jmv.23313 22711356

[78] Schenkel Jason M Masopust D. Tissue-resident memory T cells. Immunity (2014) 41(6):886–97. doi: 10.1016/j.immuni.2014.12.007 PMC4276131 25526304

[79] Masopust D Soerens AG. Tissue-resident T cells and other resident leukocytes. Annu Rev Immunol (2019) 37(1):521–46. doi: 10.1146/annurev-immunol-042617-053214 PMC7175802 30726153

[80] Varnaitė R. Adaptive immune responses to tick-borne encephalitis virus and SARS-COV-2. Sweden: Karolinska Institutet (2022).

[81] Bogovič P Lusa L Korva M Pavletič M Rus KR Lotrič-Furlan S. Inflammatory immune responses in the pathogenesis of tick-borne encephalitis. J Clin Med (2019) 8(5). doi: 10.3390/jcm8050731 PMC6571551 31121969

[82] Heinz FX Allison SL. Flavivirus structure and membrane fusion. Adv Virus Res (2003) 59:63–97. doi: 10.1016/S0065-3527(03)59003-0 14696327

[83] Agudelo M Palus M Keeffe JR Bianchini F Svoboda P Salát J. Broad and potent neutralizing human antibodies to tick-borne flaviviruses protect mice from disease. J Exp Med (2021) 218(5). doi: 10.1084/jem.20210236 PMC8040517 33831141

[84] Schosser R Reichert A Mansmann U Unger B Heininger U Kaiser R. Irregular tick-borne encephalitis vaccination schedules: the effect of a single catch-up vaccination with FSME-IMMUN. A prospective non-interventional study. Vaccine (2014) 32(20):2375–81. doi: 10.1016/j.vaccine.2014.01.072 24613521

[85] Paulke-Korinek M Kundi M Laaber B Brodtraeger N Seidl-Friedrich C Wiedermann U. Factors associated with seroimmunity against tick borne encephalitis virus 10 years after booster vaccination. Vaccine (2013) 31(9):1293–7. doi: 10.1016/j.vaccine.2012.12.075 23306371

[86] Dörrbecker B Dobler G Spiegel M Hufert FT. Tick-borne encephalitis virus and the immune response of the mammalian host. Travel Med Infect Dis (2010) 8(4):213–22. doi: 10.1016/j.tmaid.2010.05.010 20970724

[87] Loew-Baselli A Poellabauer EM Pavlova BG Fritsch S Firth C Petermann R. Prevention of tick-borne encephalitis by FSME-IMMUN vaccines: review of a clinical development programme. Vaccine (2011) 29(43):7307–19. doi: 10.1016/j.vaccine.2011.07.089 21843576

[88] Galgani I Bunge EM Hendriks L Schludermann C Marano C De Moerlooze L. Systematic literature review comparing rapid 3-dose administration of the GSK tick-borne encephalitis vaccine with other primary immunization schedules. Expert Rev Vaccines (2017) 16(9):919–32. doi: 10.1080/14760584.2017.1358620 28770638

[89] Günther G Haglund M Lindquist L Sköldenberg B Forsgren M. Intrathecal IgM, IgA and IgG antibody response in tick-borne encephalitis. Long-term follow-up related to clinical course and outcome. Clin Diagn Virol (1997) 8(1):17–29. doi: 10.1016/s0928-0197(97)00273-0 9248655

[90] Růžek D Dobler G Donoso Mantke O. Tick-borne encephalitis: pathogenesis and clinical implications. Travel Med Infect Dis (2010) 8(4):223–32. doi: 10.1016/j.tmaid.2010.06.004 20970725

[91] Holzmann H. Diagnosis of tick-borne encephalitis. Vaccine (2003) 21 Suppl 1:S36–40. doi: 10.1016/s0264-410x(02)00819-8 12628812

[92] Stiasny K Holzmann H Heinz FX. Characteristics of antibody responses in tick-borne encephalitis vaccination breakthroughs. Vaccine (2009) 27(50):7021–6. doi: 10.1016/j.vaccine.2009.09.069 19789092

[93] Taba P Schmutzhard E Forsberg P Lutsar I Ljøstad U Mygland Å. EAN consensus review on prevention, diagnosis and management of tick-borne encephalitis. Eur J Neurol (2017) 24(10):1214–e61. doi: 10.1111/ene.13356 28762591

[94] Kroschewski H Allison SL Heinz FX Mandl CW. Role of heparan sulfate for attachment and entry of tick-borne encephalitis virus. Virology (2003) 308(1):92–100. doi: 10.1016/S0042-6822(02)00097-1 12706093

[95] Pierson TC Diamond MS. Molecular mechanisms of antibody-mediated neutralisation of flavivirus infection. Expert Rev Mol Med (2008) 10:e12. doi: 10.1017/S1462399408000665 18471342 PMC2671962

[96] Mora-Cárdenas E Aloise C Faoro V Knap Gašper N Korva M Caracciolo I. Comparative specificity and sensitivity of NS1-based serological assays for the detection of flavivirus immune response. PloS Negl Trop Dis (2020) 14(1):e0008039. doi: 10.1371/journal.pntd.0008039 31995566 PMC7010293

[97] Stiasny K Leitner A Holzmann H Heinz FX. Dynamics and extent of non-structural protein 1-antibody responses in tick-borne encephalitis vaccination breakthroughs and unvaccinated patients. Viruses (2021) 13(6). doi: 10.3390/v13061007 PMC8228328 34072119

[98] Aleshin SE Timofeev AV Khoretonenko MV Zakharova LG Pashvykina GV Stephenson JR. Combined prime-boost vaccination against tick-borne encephalitis (TBE) using a recombinant vaccinia virus and a bacterial plasmid both expressing TBE virus non-structural NS1 protein. BMC Microbiol (2005) 5(1):45. doi: 10.1186/1471-2180-5-45 16076390 PMC1187892

[99] Jacobs SC Stephenson JR Wilkinson GWG. Protection elicited by a replication-defective adenovirus vector expressing the tick-borne encephalitis virus non-structural glycoprotein NS1. J Gen Virol (1994) 75(9):2399–402. doi: 10.1099/0022-1317-75-9-2399 8077939

[100] Khoretonenko MV Vorovitch MF Zakharova LG Pashvykina GV Ovsyannikova NV Stephenson JR. Vaccinia virus recombinant expressing gene of tick-borne encephalitis virus non-structural NS1 protein elicits protective activity in mice. Immunol Lett (2003) 90(2):161–3. doi: 10.1016/j.imlet.2003.09.002 14687719

[101] Kuzmenko YV Starodubova ES Shevtsova AS Chernokhaeva LL Latanova AA Preobrazhenskaia OV. Intracellular degradation and localization of NS1 of tick-borne encephalitis virus affect its protective properties. J Gen Virol (2017) 98(1):50–5. doi: 10.1099/jgv.0.000700 28221100

[102] Timofeev AV Butenko VM Stephenson JR. Genetic vaccination of mice with plasmids encoding the NS1 non-structural protein from tick-borne encephalitis virus and dengue 2 virus. Virus Genes (2004) 28(1):85–97. doi: 10.1023/B:VIRU.0000012266.04871.ce 14739654

[103] Volpina OM Volkova TD Koroev DO Ivanov VT Ozherelkov SV Khoretonenko MV. A synthetic peptide based on the NS1 non-structural protein of tick-borne encephalitis virus induces a protective immune response against fatal encephalitis in an experimental animal model. Virus Res (2005) 112(1-2):95–9. doi: 10.1016/j.virusres.2005.03.026 16022903

[104] Girl P Bestehorn-Willmann M Zange S Borde JP Dobler G von Buttlar H. Tick-borne encephalitis virus nonstructural protein 1 igG enzyme-linked immunosorbent assay for differentiating infection versus vaccination antibody responses. J Clin Microbiol (2020) 58(4). doi: 10.1128/JCM.01783-19 PMC7098735 31969423

[105] Albinsson B Rönnberg B Vene S Lundkvist Å. Antibody responses to tick-borne encephalitis virus non-structural protein 1 and whole virus antigen–a new tool in the assessment of suspected vaccine failure patients. Infect Ecol Epidemiol (2019) 9(1):1696132. doi: 10.1080/20008686.2019.1696132 31839903 PMC6896504

[106] WHO publication. Vaccines against tick-borne encephalitis: WHO position paper - Recommendations. Vaccine (2011) 29(48):8769–70. doi: 10.1016/j.vaccine.2011.07.024 21777636

[107] Holzmann H Kundi M Stiasny K Clement J McKenna P Kunz C. Correlation between ELISA, hemagglutination inhibition, and neutralization tests after vaccination against tick-borne encephalitis. J Med Virol (1996) 48(1):102–7. doi: 10.1002/(SICI)1096-9071(199601)48:1<102::AID-JMV16>3.0.CO;2-I 8825718

[108] Heinz FX Stiasny K. Flaviviruses and their antigenic structure. J Clin Virol (2012) 55(4):289–95. doi: 10.1016/j.jcv.2012.08.024 22999801

[109] Füzik T Formanová P Růžek D Yoshii K Niedrig M Plevka P. Structure of tick-borne encephalitis virus and its neutralization by a monoclonal antibody. Nat Commun (2018) 9(1):436. doi: 10.1038/s41467-018-02882-0 29382836 PMC5789857

[110] Yang X Qi J Peng R Dai L Gould EA Gao GF. Molecular basis of a protective/neutralizing monoclonal antibody targeting envelope proteins of both tick-borne encephalitis virus and louping ill virus. J Virol (2019) 93(8). doi: 10.1128/JVI.02132-18 PMC6450107 30760569

[111] Baykov IK Chojnowski G Pachl P Matveev AL Moor NA Emelianova LA. Structural insights into tick-borne encephalitis virus neutralization and animal protection by a therapeutic antibody. bioRxiv (2021). doi: 10.1101/2021.07.28.453943

[112] Tsouchnikas G Zlatkovic J Jarmer J Strauß J Vratskikh O Kundi M. Immunization with immune complexes modulates the fine specificity of antibody responses to a flavivirus antigen. J Virol (2015) 89(15):7970–8. doi: 10.1128/JVI.00938-15 PMC4505641 26018152

[113] Jarmer J Zlatkovic J Tsouchnikas G Vratskikh O Strauß J Aberle JH. Variation of the specificity of the human antibody responses after tick-borne encephalitis virus infection and vaccination. J Virol (2014) 88(23):13845–57. doi: 10.1128/JVI.02086-14 PMC4248988 25253341

[114] Heinz FX Mandl C Berger R Tuma W Kunz C. Antibody-induced conformational changes result in enhanced avidity of antibodies to different antigenic sites on the tick-borne encephalitis virus glycoprotein. Virology (1984) 133(1):25–34. doi: 10.1016/0042-6822(84)90422-7 6199892

[115] Matveev A Matveev L Stronin O Baykov I Emeljanova L Khlusevich Y. Characterization of neutralizing monoclonal antibody against tick-borne encephalitis virus in vivo. Vaccine (2020) 38(27):4309–15. doi: 10.1016/j.vaccine.2020.04.051 32409136

[116] Venturi G Martelli P Mazzolini E Fiorentini C Benedetti E Todone D. Humoral immunity in natural infection by tick-borne encephalitis virus. J Med Virol (2009) 81(4):665–71. doi: 10.1002/jmv.21431 19235849

[117] McAuley AJ Sawatsky B Ksiazek T Torres M Korva M Lotrič-Furlan S. Cross-neutralisation of viruses of the tick-borne encephalitis complex following tick-borne encephalitis vaccination and/or infection. NPJ Vaccines (2017) 2:5. doi: 10.1038/s41541-017-0009-5 29263866 PMC5627269

[118] Stiasny K Aberle JH Keller M Grubeck-Loebenstein B Heinz FX. Age affects quantity but not quality of antibody responses after vaccination with an inactivated flavivirus vaccine against tick-borne encephalitis. PloS One (2012) 7(3):e34145. doi: 10.1371/journal.pone.0034145 22461903 PMC3312914

[119] Rathore APS St John AL. Cross-reactive immunity among flaviviruses. Front Immunol (2020) 11:334. doi: 10.3389/fimmu.2020.00334 32174923 PMC7054434

[120] Mansfield KL Horton DL Johnson N Li L Barrett ADT Smith DJ. Flavivirus-induced antibody cross-reactivity. J Gen Virol (2011) 92(Pt 12):2821–9. doi: 10.1099/vir.0.031641-0 PMC3352572 21900425

[121] Chidumayo NN Yoshii K Kariwa H. Evaluation of the European tick-borne encephalitis vaccine against Omsk hemorrhagic fever virus. Microbiol Immunol (2014) 58(2):112–8. doi: 10.1111/1348-0421.12122 24329534

[122] Fritz R Orlinger KK Hofmeister Y Janecki K Traweger A Perez-Burgos L. Quantitative comparison of the cross-protection induced by tick-borne encephalitis virus vaccines based on European and Far Eastern virus subtypes. Vaccine (2012) 30(6):1165–9. doi: 10.1016/j.vaccine.2011.12.013 22178103

[123] Collins MH McGowan E Jadi R Young E Lopez CA Baric RS. Lack of durable cross-neutralizing antibodies against zika virus from dengue virus infection. Emerg Infect Dis (2017) 23(5):773–81. doi: 10.3201/eid2305.161630 PMC5403059 28418292

[124] Lindblom P Wilhelmsson P Fryland L Matussek A Haglund M Sjöwall J. Factors determining immunological response to vaccination against tick-borne encephalitis virus in older individuals. PloS One (2014) 9(6):e100860. doi: 10.1371/journal.pone.0100860 24967619 PMC4072701

[125] Bradt V Malafa S von Braun A Jarmer J Tsouchnikas G Medits I. Pre-existing yellow fever immunity impairs and modulates the antibody response to tick-borne encephalitis vaccination. NPJ Vaccines (2019) 4:38. doi: 10.1038/s41541-019-0133-5 31508246 PMC6731309

[126] Leonova GN Pavlenko EV. Characterization of neutralizing antibodies to Far Eastern of tick-borne encephalitis virus subtype and the antibody avidity for four tick-borne encephalitis vaccines in human. Vaccine (2009) 27(21):2899–904. doi: 10.1016/j.vaccine.2009.02.069 19366574

[127] Leonova GN Ternovoi VA Pavlenko EV Maistrovskaya OS Protopopova EV Loktev VB. Evaluation of vaccine Encepur Adult for induction of human neutralizing antibodies against recent Far Eastern subtype strains of tick-borne encephalitis virus. Vaccine (2007) 25(5):895–901. doi: 10.1016/j.vaccine.2006.09.014 17011677

[128] Domnich A Panatto D Arbuzova EK Signori A Avio U Gasparini R. Immunogenicity against Far Eastern and Siberian subtypes of tick-borne encephalitis (TBE) virus elicited by the currently available vaccines based on the European subtype: systematic review and meta-analysis. Hum Vaccin Immunother (2014) 10(10):2819–33. doi: 10.4161/hv.29984 PMC5443051 25483679

[129] Morozova OV Bakhvalova VN Potapova OF Grishechkin AE Isaeva EI Aldarov KV. Evaluation of immune response and protective effect of four vaccines against the tick-borne encephalitis virus. Vaccine (2014) 32(25):3101–6. doi: 10.1016/j.vaccine.2014.02.046 24631082

[130] Levanov LN Matveev LE Goncharova EP Lebedev LR Ryzhikov AB Yun TE. Chimeric antibodies against tick-borne encephalitis virus. Vaccine (2010) 28(32):5265–71. doi: 10.1016/j.vaccine.2010.05.060 20538092

[131] Matveev AL Kozlova IV Stronin OV Khlusevich YA Doroshchenko EK Baykov IK. Post-exposure administration of chimeric antibody protects mice against European, Siberian, and Far-Eastern subtypes of tick-borne encephalitis virus. PloS One (2019) 14(4):e0215075. doi: 10.1371/journal.pone.0215075 30958863 PMC6453444

[132] Baykov IK Matveev AL Stronin OV Ryzhikov AB Matveev LE Kasakin MF. A protective chimeric antibody to tick-borne encephalitis virus. Vaccine (2014) 32(29):3589–94. doi: 10.1016/j.vaccine.2014.05.012 24837772

[133] Elsterova J Palus M Sirmarova J Kopecky J Niller HH Ruzek D. Tick-borne encephalitis virus neutralization by high dose intravenous immunoglobulin. Ticks Tick Borne Dis (2017) 8(2):253–8. doi: 10.1016/j.ttbdis.2016.11.007 27884572

[134] Růžek D Dobler G Niller HH. May early intervention with high dose intravenous immunoglobulin pose a potentially successful treatment for severe cases of tick-borne encephalitis? BMC Infect Dis (2013) 13:306. doi: 10.1186/1471-2334-13-306 23822550 PMC3710210

[135] Bogovič P Lotrič-Furlan S Avšič-Županc T Korva M Lusa L Strle K. Low virus-specific igG antibodies in adverse clinical course and outcome of tick-borne encephalitis. Microorganisms (2021) 9(2). doi: 10.3390/microorganisms9020332 PMC7914885 33562267

[136] Bogovič P Lotrič-Furlan S Avšič-Županc T Lusa L Strle F. Factors associated with severity of tick-borne encephalitis: A prospective observational study. Travel Med Infect Dis (2018) 26:25–31. doi: 10.1016/j.tmaid.2018.10.003 30296483

[137] Grygorczuk S Czupryna P Pancewicz S Świerzbińska R Kondrusik M Dunaj J. Intrathecal expression of IL-5 and humoral response in patients with tick-borne encephalitis. Ticks Tick Borne Dis (2018) 9(4):896–911. doi: 10.1016/j.ttbdis.2018.03.012 29602685

[138] Toporkova MG Aleshin SE Ozherelkov SV Nadezhdina MV Stephenson JR Timofeev AV. Serum levels of interleukin 6 in recently hospitalized tick-borne encephalitis patients correlate with age, but not with disease outcome. Clin Exp Immunol (2008) 152(3):517–21. doi: 10.1111/j.1365-2249.2008.03617.x PMC2453219 18462209

[139] Veje M Studahl M Johansson M Johansson P Nolskog P Bergström T. Diagnosing tick-borne encephalitis: a re-evaluation of notified cases. Eur J Clin Microbiol Infect Dis (2018) 37(2):339–44. doi: 10.1007/s10096-017-3139-9 PMC5780526 29188467

[140] Dobler G. Diagnosis. In: Dobler G Erber W Bröker M Schmitt HJ, editors. The TBE book, 4 ed. Singapore: Global Health Press Pte Ltd (2021). p. 133–40.

[141] Hira V Rockx B. Human tick-borne encephalitis, the Netherlands. Emerg Infect Dis (2017) 23(1):169. doi: 10.3201/eid2301.161405 27938522 PMC5176219

[142] Siemieniako-Werszko A Czupryna P Moniuszko-Malinowska A Dunaj-Małyszko J Pancewicz S Grygorczuk S. Anti-TBE intrathecal synthesis as a prediction marker in TBE patients. Pathogens (2022) 11(4). doi: 10.3390/pathogens11040416 PMC9032606 35456091

[143] Kriz B Hubalek Z Marek M Daniel M Strakova P Betasova L. Results of the screening of tick-borne encephalitis virus antibodies in human sera from eight districts collected two decades apart. Vector Borne Zoonotic Dis (2015) 15(8):489–93. doi: 10.1089/vbz.2014.1747 26273810

[144] Loew-Baselli A Poellabauer EM Pavlova BG Fritsch S Koska M Bobrovsky R. Seropersistence of tick-borne encephalitis antibodies, safety and booster response to FSME-IMMUN 0.5 ml in adults aged 18-67 years. Hum Vaccin (2009) 5(8):551–6. doi: 10.4161/hv.5.8.8571 19430202

[145] Weinberger B Keller M Fischer KH Stiasny K Neuner C Heinz FX. Decreased antibody titers and booster responses in tick-borne encephalitis vaccinees aged 50-90 years. Vaccine (2010) 28(20):3511–5. doi: 10.1016/j.vaccine.2010.03.024 20332047

[146] Wittermann C Schöndorf I Gniel D. Antibody response following administration of two paediatric tick-borne encephalitis vaccines using two different vaccination schedules. Vaccine (2009) 27(10):1661–6. doi: 10.1016/j.vaccine.2008.10.003 18940221

[147] Beran J Lattanzi M Xie F Moraschini L Galgani I. Second five-year follow-up after a booster vaccination against tick-borne encephalitis following different primary vaccination schedules demonstrates at least 10 years antibody persistence. Vaccine (2019) 37(32):4623–9. doi: 10.1016/j.vaccine.2017.12.081 29397225

[148] Hainz U Jenewein B Asch E Pfeiffer KP Berger P Grubeck-Loebenstein B. Insufficient protection for healthy elderly adults by tetanus and TBE vaccines. Vaccine (2005) 23(25):3232–5. doi: 10.1016/j.vaccine.2005.01.085 15837226

[149] Rendi-Wagner P Kundi M Zent O Dvorak G Jaehnig P Holzmann H. Persistence of protective immunity following vaccination against tick-borne encephalitis–longer than expected? Vaccine (2004) 22(21-22):2743–9. doi: 10.1016/j.vaccine.2004.01.041 15246606

[150] Konior R Brzostek J Poellabauer EM Jiang Q Harper L Erber W. Seropersistence of TBE virus antibodies 10 years after first booster vaccination and response to a second booster vaccination with FSME-IMMUN 0.5mL in adults. Vaccine (2017) 35(28):3607–13. doi: 10.1016/j.vaccine.2017.03.059 28545923

[151] Jílková E Vejvalková P Stiborová I Skorkovský J Král V. Serological response to tick-borne encephalitis (TBE) vaccination in the elderly–results from an observational study. Expert Opin Biol Ther (2009) 9(7):797–803. doi: 10.1517/14712590903066711 19527104

[152] Janik M Płaczkowska S Woźniak M Bil-Lula I. Analysis of multiple risk factors for seronegative rate of anti-tick-borne encephalitis virus immunization in human serum. Medicina (Kaunas) (2020) 56(5). doi: 10.3390/medicina56050244 PMC7279439 32443896

[153] Poellabauer E Angermayr R Behre U Zhang P Harper L Schmitt HJ. Seropersistence and booster response following vaccination with FSME-IMMUN in children, adolescents, and young adults. Vaccine (2019) 37(24):3241–50. doi: 10.1016/j.vaccine.2019.03.032 30928173

[154] Schoendorf I Ternak G Oroszlàn G Nicolay U Banzhoff A Zent O. Tick-born encephalitis (TBE) vaccination in children: advantage of the rapid immunization schedule (i.e., days 0, 7, 21). Hum Vaccin (2007) 3(2):42–7. doi: 10.4161/hv.3.2.3747 17297298

[155] Beran J Douda P Gniel D Zent O. Long-term immunity after vaccination against tick-borne encephalitis with Encepur using the rapid vaccination schedule. Int J Med Microbiol (2004) 293 Suppl 37:130–3. doi: 10.1016/S1433-1128(04)80023-8 15146994

[156] Plentz A Jilg W Schwarz TF Kuhr HB Zent O. Long-term persistence of tick-borne encephalitis antibodies in adults 5 years after booster vaccination with Encepur Adults. Vaccine (2009) 27(6):853–6. doi: 10.1016/j.vaccine.2008.11.082 19071180

[157] Rendi-Wagner P Paulke-Korinek M Kundi M Wiedermann U Laaber B Kollaritsch H. Seroprotection 4 years following booster vaccination against tick-borne encephalitis. Int J Med Microbiol (2008) 298:305–8. doi: 10.1016/j.ijmm.2008.01.004

[158] Paulke-Korinek M Rendi-Wagner P Kundi M Laaber B Wiedermann U Kollaritsch H. Booster vaccinations against tick-borne encephalitis: 6 years follow-up indicates long-term protection. Vaccine (2009) 27(50):7027–30. doi: 10.1016/j.vaccine.2009.09.068 19786143

[159] Euctr PL. Open-label phase IV study to investigate the seropersistence of tick-borne encephalitis (TBE) virus antibodies after the first booster and the response to a second booster vaccination with FSME-Immun 0.5ml in adults (follow up to study 223) - TBE Seropersistence Adults (2007). Available at: https://trialsearchwhoint/Trial2aspx?TrialID=EUCTR2007-000440-27-PL.

[160] Vene S Haglund M Lundkvist A Lindquist L Forsgren M. Study of the serological response after vaccination against tick-borne encephalitis in Sweden. Vaccine (2007) 25(2):366–72. doi: 10.1016/j.vaccine.2006.07.026 16959384

[161] Schöndorf I Schönfeld C Nicolay U Zent O Banzhoff A. Response to tick-borne encephalitis (TBE) booster vaccination after prolonged time intervals to primary immunization with the rapid schedule. Int J Med Microbiol (2006) 296 Suppl 40:208–12. doi: 10.1016/j.ijmm.2006.01.009 16531118

[162] Aerssens A Cochez C Niedrig M Heyman P Kühlmann-Rabens I Soentjens P. Analysis of delayed TBE-vaccine booster after primary vaccination. J Travel Med (2016) 23(2):tav020. doi: 10.1093/jtm/tav020 26858269

[163] Rendi-Wagner P Zent O Jilg W Plentz A Beran J Kollaritsch H. Persistence of antibodies after vaccination against tick-borne encephalitis. Int J Med Microbiol (2006) 296 Suppl 40:202–7. doi: 10.1016/j.ijmm.2006.01.030 16524776

[164] Rendi-Wagner P Kundi M Zent O Banzhoff A Jaehnig P Stemberger R. Immunogenicity and safety of a booster vaccination against tick-borne encephalitis more than 3 years following the last immunisation. Vaccine (2004) 23(4):427–34. doi: 10.1016/j.vaccine.2004.07.002 15530690

[165] Rendi-Wagner P Paulke-Korinek M Kundi M Wiedermann U Laaber B Kollaritsch H. Antibody persistence following booster vaccination against tick-borne encephalitis: 3-year post-booster follow-up. Vaccine (2007) 25(27):5097–101. doi: 10.1016/j.vaccine.2007.01.116 17555850

[166] Wittermann C Izu A Petri E Gniel D Fragapane E. Five year follow-up after primary vaccination against tick-borne encephalitis in children. Vaccine (2015) 33(15):1824–9. doi: 10.1016/j.vaccine.2015.02.038 25728316

[167] Beran J Xie F Zent O. Five year follow-up after a first booster vaccination against tick-borne encephalitis following different primary vaccination schedules demonstrates long-term antibody persistence and safety. Vaccine (2014) 32(34):4275–80. doi: 10.1016/j.vaccine.2014.06.028 24950352

[168] Altpeter ES Zimmermann H Oberreich J Peíter O Dvořák C. Tick related diseases in Switzerland, 2008 to 2011. Swiss Med weekly (2013) 143:w13725. doi: 10.4414/smw.2013.13725 23299974

[169] Schuler M Zimmermann H Altpeter E Heininger U. Epidemiology of tick-borne encephalitis in Switzerland, 2005 to 2011. Euro Surveill (2014) 19(13). doi: 10.2807/1560-7917.ES2014.19.13.20756 24721541

[170] Lotrič-Furlan S Bogovič P Avšič-Županc T Jelovšek M Lusa L Strle F. Tick-borne encephalitis in patients vaccinated against this disease. J Intern Med (2017) 282(2):142–55. doi: 10.1111/joim.12625 28440879

[171] Hansson KE Rosdahl A Insulander M Vene S Lindquist L Gredmark-Russ S. Tick-borne encephalitis vaccine failures: A 10-year retrospective study supporting the rationale for adding an extra priming dose in individuals starting at age 50 years. Clin Infect Dis (2020) 70(2):245–51. doi: 10.1093/cid/ciz176 PMC6938976 30843030

[172] Kantele A Rombo L Vene S Kundi M Lindquist L Erra EO. Three-dose versus four-dose primary schedules for tick-borne encephalitis (TBE) vaccine FSME-immun for those aged 50 years or older: A single-centre, open-label, randomized controlled trial. Vaccine (2022) 40(9):1299–305. doi: 10.1016/j.vaccine.2022.01.022 35101266

[173] Heinz FX Holzmann H Essl A Kundi M. Field effectiveness of vaccination against tick-borne encephalitis. Vaccine (2007) 25(43):7559–67. doi: 10.1016/j.vaccine.2007.08.024 17869389

[174] Heinz FX Stiasny K Holzmann H Grgic-Vitek M Kriz B Essl A. Vaccination and tick-borne encephalitis, central Europe. Emerg Infect Dis (2013) 19(1):69–76. doi: 10.3201/eid1901.120458 23259984 PMC3557984

[175] Erber W Khan F Zavadska D Freimane Z Dobler G Böhmer MM. Effectiveness of TBE vaccination in southern Germany and Latvia. Vaccine (2022) 40(5):819–25. doi: 10.1016/j.vaccine.2021.12.028 34952753

[176] Zens KD Haile SR Schmidt AJ Altpeter ES Fehr JS Lang P. Retrospective, matched case-control analysis of tickborne encephalitis vaccine effectiveness by booster interval, Switzerland 2006-2020. BMJ Open (2022) 12(4):e061228. doi: 10.1136/bmjopen-2022-061228 PMC9036433 35459683

[177] Nygren TM Pilic A Böhmer MM Wagner-Wiening C Wichmann O Harder T. Tick-borne encephalitis vaccine effectiveness and barriers to vaccination in Germany. Sci Rep (2022) 12(1):11706. doi: 10.1038/s41598-022-15447-5 35810184 PMC9271034

[178] Kunz C. TBE vaccination and the Austrian experience. Vaccine (2003) 21 Suppl 1:S50–5. doi: 10.1016/S0264-410X(02)00813-7 12628814

[179] Baroutsou V Zens KD Sinniger P Fehr J Lang P. Analysis of Tick-borne Encephalitis vaccination coverage and compliance in adults in Switzerland, 2018. Vaccine (2020) 38(49):7825–33. doi: 10.1016/j.vaccine.2020.10.022 33164805

[180] Zens KD Baroutsou V Sinniger P Lang P. A cross-sectional study evaluating tick-borne encephalitis vaccine uptake and timeliness among adults in Switzerland. PloS One (2021) 16(12):e0247216. doi: 10.1371/journal.pone.0247216 34905534 PMC8670666

[181] Angulo FJ Zhang P Halsby K Kelly P Pilz A Madhava H. A systematic literature review of the effectiveness of tick-borne encephalitis vaccines in Europe. Vaccine (2023) 41(47):6914–21. doi: 10.1016/j.vaccine.2023.10.014 37858450

[182] Miazga W Wnuk K Tatara T Świtalski J Matera A Religioni U. The long-term efficacy of tick-borne encephalitis vaccines available in Europe - a systematic review. BMC Infect Dis (2023) 23(1):621. doi: 10.1186/s12879-023-08562-9 37735357 PMC10515056

[183] Beauté J Spiteri G Warns-Petit E Zeller H. Tick-borne encephalitis in Europe, 2012 to 2016. Euro Surveill (2018) 23(45). doi: 10.2807/1560-7917.ES.2018.23.45.1800201 PMC6234529 30424829

[184] Andersson CR Vene S Insulander M Lindquist L Lundkvist A Günther G. Vaccine failures after active immunisation against tick-borne encephalitis. Vaccine (2010) 28(16):2827–31. doi: 10.1016/j.vaccine.2010.02.001 20167301

[185] Schmidt AJ Altpeter E Graf S Steffen R. Tick-borne encephalitis (TBE) in Switzerland: does the prolongation of vaccine booster intervals result in an increased risk of breakthroughs? J Travel Med (2022) 29(2). doi: 10.1093/jtm/taab158 34581402

